# Common Problems! and Common Solutions? — Teaching at the Intersection Between Public Health and Criminology: A Public Health Perspective

**DOI:** 10.5334/aogh.4375

**Published:** 2024-02-12

**Authors:** Gloria Macassa, Cormac McGrath

**Affiliations:** 1 Department of Public Health and Sports Science, Faculty of Occupational and Health Sciences, University of Gävle, Kungsbacksvägen 47, 80176 Gävle, Sweden; 2 Department of Public Health, School of Health Sciences, University of Skövde, 541 28 Skövde, Sweden; 3 EPIUnit–Instituto de Saude Publica, Universidade do Porto, Rua das Taipas 135, 4050–600 Porto, Portugal; 4 Department of Education, Stockholm University, 10691 Stockholm, Sweden

**Keywords:** causation, intersection public health–criminology, prevention, social determinants, sustainable development

## Abstract

Public health and criminology share similar current and future challenges, mostly related to crime and health causation, prevention, and sustainable development. Interdisciplinary and transdisciplinary approaches to education at the intersection of public health and criminology can be an integral part of future training in areas of mutual interest. Based on reflections on teaching criminology students, this viewpoint discusses the main interconnections between public health and criminology teaching through the public health lens. The paper discusses potential challenges associated with interdisciplinarity and transdisciplinarity. Among these challenges is communication across the different fields and their perspectives to be able to achieve the desired complementarity at the intersection of the two disciplines.

## 1. Introduction

Today, societies are experiencing a myriad of challenges, ranging from urbanization and migration to climate change, inequality, violence, and crime. Efforts to address these challenges require inter- and transdisciplinary approaches in education to foster a common understanding of causes and prevention [1]. Contrary to multidisciplinary approaches, where disciplines remain intact but simultaneously focus on a theme of interest [1,2], interdisciplinary approaches conjoin disciplines to develop a shared understanding of a selected theme or solution to a problem [3,4]. Moreover, transdisciplinary approaches foster collaboration around real-world problems without regard to discipline-specific understandings. It is argued that transdisciplinary learning entails the exploration of a relevant concept, issue, or problem that integrates the perspectives of multiple disciplines to connect new knowledge and a deeper understanding of real-life experiences [5,6,7,8,9,10].

Based on the first author’s reflections (GM; teacher and researcher in public health and epidemiology) on teaching criminology students in a bachelor program in applied criminology at a large European university (in East Central Sweden), this viewpoint identifies the main connections between public health and criminology through a public health lens. First, the viewpoint briefly identifies the connections between crime and public health. Then, it elaborates on the common areas of intersection between public health and criminology. Finally, it concludes by discussing the opportunities and challenges of interdisciplinary and transdisciplinary approaches to education.

## 2. Crime and Public Health

Violence and crime are growing concerns and represent public health problems worldwide, regardless of level of development [11]. Most recently, the 2022 INTERPOL Global Crime Trend report stated that global organized crime was among the top ten crimes perceived to be a high or very high threat across member states, as was illicit production and distribution of synthetic drugs and cannabis trafficking [12]. Furthermore, recidivism rates are increasing and most likely are due to the persistent socioeconomic disparities and neighborhood deprivation encountered by those who return to their communities after serving a prison sentence, as well as issues related to the effectiveness of post-release offender management programs [13]. Crime has an impact on health and wellbeing, and evidence has consistently shown that it is associated with an array of physical and mental health outcomes across a variety of contexts [14]. Conversely, mental health can have an impact on crime [14]. Specifically, and in relation to mental health, it is known that people who suffer from mental illness (including those with substance use-related disorders) are overrepresented in the criminal justice system [15]. It is suggested that this can be a result of the interconnection of several factors related to structural poverty, stigma, racism, inadequate housing, and a history of trauma, co-occurring substance abuse, and mental health problems [16]. Moreover, others point out that mental health illnesses can result in behaviors that might call attention to criminal justice responses [15]. Furthermore, lack of access to mental health care (in terms of treatment and support) can be a contributing factor to the criminalization of people with mental illnesses [17]. For instance, Hartford et al. argue that this occurs because individuals with mental illness who do not get adequate treatment and support have a high likelihood of committing crimes that warrant police intervention [17].

## 3. The Intersection Between Public Health and Criminology

There are five areas of potential intersection between public health and criminology (as conceptualized by the first author): (1) social determinants (the root causes of health and criminal behavior/crime); (2) epidemiological criminology (Epi-Crim); (3) intervention and experimentation; (4) the prevention approach (the public health approach to crime); and (5) sustainable development and safety. These areas were taught in courses in the first (Introduction to Criminology and Crime Prevention) and second (Criminology Theory) years of the program.

### 3.1. Social determinants of health and criminal behavior

Both public health and criminology are assumed to be interdisciplinary/transdisciplinary by nature, but they remain predominantly discipline-based. As new challenges emerge regarding the root causes of criminal behavior as well as crime prevention, these disciplines need to find a way to target common problems together with a range of other disciplines (e.g., law, social work, psychology, psychiatry, etc.) [18,19,20]. Crime in communities and society at large can have a long-lasting impact both on the lives of the victims, perpetrators, and witnesses and on the adjacent society [18,19,20]. In addition, crime can add to a compounding array of factors that are likely to impact the social determinants of health for people living in a community [18].

According to the World Health Organization’s (WHO) Commission on the Social Determinants of Health (CSDH), the social determinants of health are the conditions in which people are born, grow, work, live, and age, and the wider set of forces and systems (economic policies and systems, social policies, social norms, developmental agendas, and political systems) shaping the conditions of their daily lives [21,22]. Some have argued that these determinants are root causes of criminal behavior/crime [23,24,25,26,27]. For instance, Caruso argues that when public health institutions identify and act on the social determinants of health to address health inequalities (and inequities), this should mean equally identifying the social determinants of criminal behavior (SDCBs) to reduce crime and improve public safety [24]. Furthermore, he lists poverty, socioeconomic status, abuse and domestic violence, housing, mental illnesses and health care, education, the environment, and nutrition, as well as neuroscience and psychopathy, as the main common social determinants [24].

Applying a social determinants perspective to criminal behavior and crime entails introducing the notion that there are social inequalities (and inequities) in crime, which means that there are differences (i.e., a social gradient) in the distribution of the material conditions and other factors that contribute to criminal behavior and crime, and that some of these differences are unjust and unfair [23]. Therefore, inequalities that are unjust and unfair (i.e., inequities) can be tackled by joint prevention and intervention programs addressing inequalities and inequities in health and crime [24].

### 3.2. Epidemiological criminology

A sub-discipline of epidemiology, Epi-Crim, is defined as an emerging field that seeks to address, understand, and synthesize the fields of epidemiology and criminology to drive a greater understanding of the causes of crime, as well as viewing poor health states as the core etiology of criminal behavior [18,19,20, 28,29,30]. Moreover, it is argued that Epi-Crim uses traditional public health principles (epidemiology) to understand the causes of crime and criminal justice-related concerns such as substance use, violence, and crime [31]. Potter and Akers likewise state that Epi-Crim epistemologically and aetiologically need to be seen as an integration of the theories, methods, practices, and technologies that are used in both public health and criminal justice, incorporated into an interdisciplinary framework of epidemiology and criminology [28]. The framework includes biological, psychological, and social factors—a bio-psycho-social approach [28] or sociobiological determinism [32]. In addition, for Potter and Akers, the Epi-Crim framework enables focusing on the study of dynamic determinants that contribute both to criminogenic risks and health risks (e.g., social, economic, legal, geospatial, and environmental) [28]. Furthermore, Epi-Crim allows both epidemiologists and criminologists to seek to uncover the reasons behind crime and the consequences it has for victims and offenders. Epi-Crim may also lead to crime reduction through evidence-based interventions and experiments [33] in a manner that neither discipline traditionally would do. Historically, epidemiology has centered on causation [34] and the health consequences for victims [35] as compared to criminology, which has paid more attention to offenders [36]. Regarding the role of experimental epidemiology in aiding evidence, Branas argued that the discipline needed to continue to strive and carry experiments to implementation (as compared to earlier stages where observation studies were the majority) as a way to provide complete evidence around solutions to contemporary complex health problems [37] (as well as complex health-related states such as crime) [24, 38].

### 3.3. Intervention and experimentation

Interventions and experiments are important at the intersection of public health and criminology. Guided by the searching for causation facilitated by Epi-Crim, interventions and experiments can play an important role in investigating the determinants of health inequalities (and inequities) in crime (e.g., victimization and perpetration) as well as the identification of effective interventions that are of paramount importance to crime reduction [18, 20]. However, although many public health issues (including crime) are complex and require preventive actions at different levels (e.g., biological, socioeconomic, and environmental levels to improve population-level outcomes), many studies, including current ones, still lack complex, multi-level, population-level intervention evidence (and more specifically, the use of natural experiments as an intervention tool). For instance, Ridgeway points out that although in the United States, crime and the justice system are expensive and spend around $280 billion per year on police, courts, and corrections [39], only now are criminologists using more statistical methods that include experimental designs and natural experiments to improve their understanding of crime and the criminal justice system’s response to crime [39]. He refers to examples of studies that were being carried out by criminologists (to answer criminological questions), such as switches to and from daylight saving time to study racial bias, quirks in grant funding formulas to study the effect of police on crime, and natural disasters to study the effect of relocation on desistance from crime [39]. On the other hand, the Andersen and Hyatt review of randomized experiments in Scandinavian criminal justice show that in Europe (Denmark, Norway, and Sweden), which have different penal philosophies than the US and a tradition of focusing on rehabilitation, only eight experiments with an offending or delinquency outcome were published before 2015, of which six focused primarily on medical or psychological treatments and argued that such distribution was the result of the unique regional epistemological traditions [40]. Criminologists and public health researchers are expected to increase their use of both interventions and experiments to provide robust evidence of what works best to reduce criminal behavior and crime [39,40]. The integration of intervention and experimentation approaches from epidemiology (public health) and criminology may result in a greater theoretical advancement in the understanding of the causes of lethal violence and in practical applications that will help reduce the excess morbidity and mortality due to violence and the public health burden it presents [18,19,20, 24, 33].

### 3.4. The public health approach to crime prevention

Because public health and criminology share several key determinants and can, within the framework of Epi-Crim, identify the root causes of crime and ill health, they can logically be complementary in prevention. In recent years, there has been an increase in voices arguing for the use of the “public health approach to crime prevention” instead of relying solely on criminal justice prevention [41,42,43,44,45]. For instance, public health prevention aims at primordial and primary prevention. This includes policies and programs that universally address social determinants of health (and criminal behavior), which are environmental, social, and economic factors, such as physical and psychological safety and social wellbeing, to promote health for everyone, as well as recognizing the structural issues (e.g., discrimination, racism, and economic inequality) that are barriers to full health [21]. For instance, it is argued that ecological context matters (e.g., families, institutions, schools, workplaces, communities, and societies) [27]. Furthermore, a public health approach to crime prevention will act to remedy health disparities by recognizing the need for intersectoral collaboration. Such an approach requires community engagement as much as it requires public health-specific prevention approaches [46,47]. It is argued that the public health approach to crime prevention can be carried out side by side with the traditional crime justice approach, which has long relied on arrest, prosecution, surveillance, and incapacitation of offenders, with limited focus on rehabilitation or reintegration [43,44]. Also, the public health approach to prevention includes primary, secondary, and tertiary prevention. For instance, primary crime prevention is aimed at stopping crime before it occurs (it focuses on social, economic, environmental, and situational factors, such as strengthening community structural and socioeconomic factors). In public health, primary prevention refers to all actions directed to avoid the manifestation of a disease (including actions to improve health through changing the impact of social and economic determinants on health, the provision of information on behavioral and medical health risks across all ages, etc.). Secondary crime prevention engages in early identification of potential offenders and seeks to intervene in their lives in such a way that they never commit criminal violation [43]; in public health, secondary prevention emphasizes early disease detection (e.g., through screening), and its target is healthy-appearing individuals with subclinical forms of disease [48,49]. Tertiary crime prevention deals with actual offenders and involves intervention in their lives in such a fashion that they will not commit further offenses [48]. In public health, tertiary prevention is aimed at preventing successive incidents after disease has occurred in order to reduce the occurrence as well as the severity of sequelae [49].

Despite the potential advantages of a public health approach to crime prevention (with a focus on intersectoral collaboration), there are challenges to be considered, such as mission drift, financial costs, difficulties in evaluating results, and perceived loss of organizational autonomy and legitimacy (for both public health and criminal justice organizations) [50,51]. However, it is argued that the potential drawbacks can be solved through communication [52]. Also, the practical integration of public health, criminology, and criminal justice into crime prevention strategies will need to be adapted to the actual context in which it takes place. This is because there are differences in the criminal justice systems across countries (in terms of courts, the police, and correction systems) [53]. For instance, using an example of the European and United States prison systems, Kovacevic and Settle showed that these differences had an influence on recidivism rates. Kovacevic and Settle argue that the US prison system relied heavily on punishment and public safety, while the European system, specifically that of Northern Europe, focused more on rehabilitation, where perpetrators were less prone to recidivism as compared to those in US prisons [53]. This is in line with the argument made by Potter and Rosky on the practical challenges of integrating public health, criminology, and criminal justice approaches to crime prevention. In their seminal paper entitled “The iron fist in the latex glove: the intersection of public health and criminal justice,” they argue that making an academic link between public health and criminology (criminal justice) was more straight-forward than in practice, especially in terms of evidence-based interventions supporting policy and street-level integration [26]. Still, that challenge prevails today, given that there is a scarcity of rigorous interventions and experimental studies that have used the public health approach to crime prevention (specifically on what works or not). However, the scant evidence emerging from US-based studies is promising as it points out that the public health approach can serve as complementary when integrated with policing strategies to reduce crime [54]. For instance, in the past decade or so, cure violence (CV) has been one of the most commonly used public health models to reduce gun violence in US cities. It is defined as an approach to reducing gun violence that seeks to change individual and community norms about violence and crime [55,56]. According to Butts et al., CV programs rely on three main elements to stop the transmission of violent behaviors: (i) interrupting transmission directly; (ii) identifying and changing the thoughts of potential transmitters (e.g., people at a higher risk of perpetrating violence); and (iii) changing norms regarding violence [55]. Although studies carried out in US [57,58,59,60,61] as well as elsewhere [62] have shown some effectiveness in crime reduction, a recent study by Buggs and colleagues that estimated the long-term impact of Safe Streets Baltimore, a CV outreach and violence interruption model on firearm violence, found that it produced disparate impacts on violent crime across implementation sites with more evidence of harm than benefit [61]. The authors emphasized the need to identify and replicate components of effective community outreach and violence interruption work as well as to directly address systemic drivers of community violence as a means to be able to reduce violence in communities nationwide. Nonetheless, an experimental study carried out in New York City that aimed to test the influence of two interventions on violence: (1) cure violence and (2) directed police patrol in violence hot spots; Cerda and colleagues found that with no intervention, 3.87% (95% CI, 3.84, 3.90) of agents were victimized per year and that implementing the violence interrupter intervention for 10 years decreased victimization by 13% (to 3.35% [3.32, 3.39]). Implementing hot-spots, policing, and doubling the police force for 10 years reduced annual victimization by about 11% (to 3.46% [3.42, 3.49]) and increasing the police force by 40%, combined with implementing the violence interrupter intervention for 10 years, decreased violence by 19% (to 3.13% [3.09, 3.16]) [54]. The authors concluded their study by stating that combining investment in a public health, community-based approach to violence prevention, and a criminal justice approach focused on deterrence achieved more to reduce population-level rates of urban violence than either could do in isolation [54].

### 3.5. Criminology and public health share links to sustainable development

Both criminology and public health share links to sustainable development through the safety of communities and societies [63,64,65,66,67]. Safety contributes both to wellbeing and to improved quality of life [68,69]. It is suggested that a society undergoes sustainable development only when there are no apparent threats to its evolution and there are sufficient operational conditions [68,69]. In addition, it is argued that the main threats to public security include terrorism, organized crime, drug and arms smuggling, cyber threats, trafficking in humans and their organs, and gang violence, which all have an impact on population health and wellbeing. Moreover, the United Nations Sustainable Development Goals agenda includes Goal 16, which is about promoting peaceful and inclusive societies, providing access to justice for all, and building effective, accountable, and inclusive institutions at all levels [70]. Goal 16 targets 16.1: Significantly reducing all forms of violence and related death rates everywhere; 16.2: Ending abuse, exploitation, trafficking, and all forms of violence against and torture of children; and 16.4: Significantly reducing illicit financial and arms flows, strengthening the recovery and return of stolen assets, and combatting all forms of organized crime by 2030 [71]. For instance, in a recent study, Hoefler and colleagues suggest that to monitor progress towards the achievement of peaceful and sustainable societies and sustainable development goal 16 in particular, accurate estimates of violence related deaths (target 1) and conflict-related deaths (sub-target 16.1.2) there is a need to have more accurate estimates of violence related deaths [72]. Furthermore, Hoeffler and colleagues propose an enlargement of sub-target 16.1.2 to cover all other forms of collective violence (and not only conflict-related deaths) to avoid a double count of cases, which will require refining the definition and measurement of collective violence that will, in turn, facilitate countries as well as the global monitoring of progress towards the achievement of peaceful and sustainable societies [72]. This proposition by Hoeffler and colleagues is of importance for the intersection between public health and criminology, given their mutual interest in monitoring crime and its consequences.

## 4. Discussion and Conclusion

There are both opportunities and challenges when taking an interdisciplinary and transdisciplinary approach to teaching at the intersection of public health and criminology. The five areas outlined above are used to implement interdisciplinary and transdisciplinary teaching at the nexus between public health and criminology (Bachelor of Applied Criminology). Interdisciplinary and transdisciplinary teaching has benefits specifically for issues related to causality (in the search for the root causes of crime and health) as well as prevention of crime and ill health. It is argued that interdisciplinary teaching can help students develop critical thinking, foster creativity, improve communication skills, and provide them with an in-depth understanding of complex issues and connections [73]. Similarly, a transdisciplinary approach enables a comprehensive perspective on people’s perceptions of their lives and gives insights into different levels of knowledge and reality, as the link between the abstract dimension and the concrete dimension of experience is demonstrated [73]. It is important to teach criminology students that the social determinants of health, which can help explain the social gradient of health and wellbeing, are also important determinants of criminal behavior, and that action on one would benefit the other [27, 74]. And as suggested by the Cerda study, potential interventions aimed at reducing crime are likely to have major benefits when carried out through interdisciplinary and transdisciplinary lenses [54]. Some have argued that interdisciplinary and transdisciplinary approaches entail addressing challenges through the problematization of issues, problem identification, and problem solving of situations and dilemmas in a way that incites the natural curiosity of students. This in turn motivates and inspires learning through engagement with authentic problems [73]. Transdisciplinarity is seen as a preferred alternative to the “dualistic perspective” of viewing the world through separate disciplines, thus operating in silos and ignoring the potential gray areas between them [75,76,77]. Transdisciplinarity goes beyond duality to promote interconnectivity, which includes the potential gray areas between disciplines [77]. Regarding the areas of interconnection suggested above, this would mean identifying risk and protective factors within the social determinants of health and criminal behavior using epidemiological methods, as well as applying the results to aid strategies aimed at crime prevention based on the public health approach. Some argue that transdisciplinarity is the most appropriate approach for addressing global challenges, especially society’s so-called “wicked problems” and the global increase in crime [78]. It is important to discuss with students the ongoing debate in many countries about the need for collaboration between criminal justice and public health professionals in crime prevention and intervention.

However, there are known barriers to interdisciplinarity and transdisciplinarity that will need to be considered when attempting to teach at the intersection of public health and criminology. It is suggested that interdisciplinarity can be complex, owing to difficulties arising from the need for communication across the different fields and their perspectives and from the potential incompatibility of theories and methods. It is also difficult because of limited funding and financial resources [78,79]. In a conceptual literature review on transdisciplinary collaboration, McGregor identified four main challenges: (a) managing group processes; (b) reflexivity; (c) common learning processes; and (d) facilitating integration and synthesis. She argued that these challenges reflect the special qualities of transdisciplinary collaboration, where individual as well as collective diversities can deeply affect transdisciplinary collaboration [79]. Extrapolating from Lawrence et al., we acknowledge that other challenges relate to students’ ability to be proficient in both disciplines when engaged in learning in transdisciplinary programs, as well as their difficulty in understanding the impact of transdisciplinarity in practice settings [80]. Despite these barriers to both interdisciplinarity and transdisciplinarity in education and learning, it is still worthwhile to pursue this approach, especially when there is an important intersectionality between the two disciplines, as in the case of public health and criminology. This is crucial considering the current debate regarding violence and crime prevention, reflected in terms such as “public health approach to crime prevention” or “public health approach to policing” across many countries and continents [48, 81,82].

Interdisciplinary and transdisciplinary approaches to public health and criminology have practical implications for research. For instance, research on crime and health causation as well as prevention can help reduce inequities in crime and ill health across different contexts [83,84,85,86]. Furthermore, public health and criminology can together contribute to tackling sustainable development challenges needing cooperation, such as urbanization and safety, as well as climate-related threats to crime and health [87,88]. As criminology needs to respond to the change in crime due to, for example, advances in technology (crime exponentiation) in terms of crime accessibility, productivity, and diversification [89], public health can be one of the needed partners in the future to better understand the impact of cybercrime (e.g., fraud, scams) on population health and wellbeing [90,91].
